# Antifungal pulse therapy for onychomycosis: a narrative review

**DOI:** 10.1007/s00228-026-04187-4

**Published:** 2026-09-25

**Authors:** Anushka Koshy, Ashwin Kamath

**Affiliations:** Department of Pharmacology, Kasturba Medical College Mangalore, Manipal Academy of Higher Education, Manipal, India

**Keywords:** Onychomycosis, Pulse therapy, Terbinafine, Itraconazole, Antifungal agents, Drug safety

## Abstract

**Purpose:**

Onychomycosis is a common chronic fungal infection of the nail unit that may be associated with significant functional impairment and impaired quality of life. Systemic antifungal drugs remain the cornerstone of treatment for moderate-to-severe disease, with continuous oral terbinafine and pulse itraconazole being the most widely used systemic antifungals. Pulse regimens involve intermittent administration of relatively high doses of antifungals with the aim of reducing cumulative drug exposure, treatment costs, and treatment burden while maintaining efficacy. However, the comparative effectiveness of pulse and continuous regimens remains unclear. This narrative review summarizes the current evidence regarding antifungal pulse therapy for onychomycosis.

**Methods:**

A literature search was conducted using PubMed and Scopus databases to identify relevant randomized trials, comparative studies, systematic reviews, meta-analyses, observational studies, and case reports/case series published between March 2001 and April 2026. Evidence relating to treatment regimens, pharmacologic rationale, efficacy, and safety was reviewed and synthesized.

**Results:**

The available evidence suggests that pulse therapy is an effective treatment option for onychomycosis. Among the currently available pulse regimens, itraconazole has the strongest supporting evidence and has demonstrated clinical and mycological outcomes comparable to continuous therapy in selected patient populations. Modified pulse schedules, prolonged regimens, and combination approaches incorporating topical antifungal agents have also shown promising results. In contrast, evidence supporting pulse terbinafine is less consistent, with several randomized controlled trials favoring continuous terbinafine, particularly in dermatophyte toenail onychomycosis. Fluconazole administered in weekly regimens appears to be less effective than terbinafine- or itraconazole-based regimens and is generally considered a second-line option. From a safety perspective, pulse and continuous regimens demonstrate safety profiles comparable to those of continuous therapy, with gastrointestinal adverse effects being the most frequently reported events.

**Conclusion:**

The current evidence supports continuous terbinafine as the preferred first-line systemic treatment for dermatophyte onychomycosis, whereas pulse itraconazole remains an important alternative for patients who are unable to tolerate terbinafine, have contraindications to its use, or have failed previous therapy. Further well-designed randomized studies with standardized outcome measures and long-term assessment of recurrence are required to better define the optimal role of pulse antifungal therapy in onychomycosis management.

## Introduction

Onychomycosis is a common fungal infection of the nail unit involving the nail plate, nail bed, and/or nail matrix. It is caused predominantly by dermatophytes, particularly *Trichophyton rubrum* and *Trichophyton interdigitale (T. mentagrophytes)*, although non-dermatophyte molds and yeasts may also be involved. Toenails are affected more frequently than fingernails [1]. The global prevalence of onychomycosis is estimated to be approximately 5.5%, with a higher prevalence reported among males [2–4]. Established risk factors for onychomycosis include increasing age, diabetes mellitus, immunosuppression, peripheral vascular disease, exposure to humid environments, frequent use of communal facilities such as gyms and swimming pools, and the use of occlusive footwear [5–7].

Besides the physical impairment, onychomycosis can have a substantial impact on the quality of life of the affected individual. Nail discoloration, thickening, and deformity may result in pain, discomfort, and impairment of daily activities, while the visible nature of the disease can contribute to embarrassment, social anxiety, and reduced self-esteem [8]. Effective treatment is therefore important not only to achieve mycological cure but also to improve quality of life, prevent disease progression, reduce recurrence, and minimize the overall burden of treatment.

Current therapeutic options include topical and systemic antifungal agents, used either alone or in combination, depending on the disease severity and extent of nail involvement. Oral terbinafine and itraconazole remain the most widely used systemic agents; topical therapies such as efinaconazole, ciclopirox, and amorolfine are commonly employed in patients with limited disease or when systemic therapy is unsuitable [9–13]. Fosravuconazole, an oral prodrug of ravuconazole with better bioavailability, has been approved in Japan as a daily regimen, recommending it over itraconazole pulse therapy, although direct comparative evidence is lacking [14, 15]. Alternative approaches, including laser-based therapies, nail drilling, and surgical interventions, may be considered in selected cases [16–20].

Among the systemic treatment strategies, pulse therapy has attracted considerable interest because it aims to reduce cumulative drug exposure while maintaining therapeutic efficacy. Pulse regimens involve intermittent administration of relatively high doses of antifungal drugs, taking advantage of the prolonged persistence of certain antifungals within the nail plate [21–24]. Potential advantages include improved convenience, reduced treatment costs, and lower cumulative exposure to systemic medication. However, the comparative effectiveness of pulse and continuous regimens remains unclear. While several studies have reported comparable outcomes between the two approaches [25, 26], others have demonstrated superior cure rates with continuous therapy, particularly continuous terbinafine therapy in dermatophyte toenail onychomycosis [27]. These conflicting findings contribute to the uncertainty regarding the optimal role of pulse therapy in routine clinical practice.

Evidence from systematic reviews and long-term follow-up studies generally supports continuous terbinafine therapy as the most effective oral treatment for dermatophyte toenail onychomycosis. A Cochrane review reported superior clinical and mycological cure rates with oral terbinafine compared with azole antifungals, although pulse regimens were not specifically evaluated [28]. Similarly, a 5-year prospective blinded follow-up study demonstrated superior long-term outcomes with continuous terbinafine compared with intermittent itraconazole in dermatophyte toenail onychomycosis [29]. Accordingly, contemporary guidelines, including those from the British Association of Dermatologists and the Nail Society of India, recommend continuous terbinafine as the first-line therapy for most cases of dermatophyte onychomycosis [30, 31]. Pulse itraconazole remains an important alternative for patients who are unable to tolerate terbinafine, have contraindications to its use, or have failed previous therapy, and it may be particularly useful in selected non-dermatophyte mold infections [30]. Although the differences in adverse event rates between continuous terbinafine and pulse itraconazole appear minimal [28], pulse regimens may offer practical advantages in selected patients. Topical therapy remains an appropriate first-line option for children and adults with mild or limited disease, whereas oral terbinafine remains preferred in most patients requiring systemic treatment [30, 32].

Given the widespread use of pulse regimens and the continuing uncertainty regarding their comparative efficacy, safety, and place in therapy, a critical appraisal of the available evidence is warranted. This narrative review summarizes currently used pulse antifungal regimens for onychomycosis, discusses their pharmacologic rationale, and evaluates the available evidence regarding their efficacy and safety.

## Literature search

A literature search was conducted using the PubMed and Scopus databases to identify studies evaluating pulse antifungal therapy for onychomycosis. Relevant randomized controlled trials, comparative clinical studies, systematic reviews, meta-analyses, observational studies, and informative case reports/case series were considered for inclusion. Only articles published in the English language were included. The literature search covered publications from March 2001 to April 2026. The period from March 2001 onward was selected to present evidence most relevant to contemporary clinical practice; earlier publications were considered where necessary to explain the pharmacological basis of pulse therapy.

The following search strategy was used in PubMed: (“Onychomycosis“[Mesh] OR onychomycosis[tiab] OR onychomycoses[tiab] OR “tinea unguium“[tiab] OR “fungal nail infection“[tiab]) AND (antifungal*[tiab] OR “Antifungal Agents“[Mesh] OR terbinafine[tiab] OR itraconazole[tiab] OR fluconazole[tiab]) AND (“pulse therapy“[tiab] OR “pulse regimen“[tiab] OR “pulse terbinafine“[tiab] OR “pulse itraconazole“[tiab] OR “continuous therapy“[tiab] OR “continuous terbinafine“[tiab] OR “continuous itraconazole“[tiab]) AND (efficacy[tiab] OR “treatment outcome“[Mesh] OR “adverse effects“[tiab] OR safety[tiab] OR “adverse events“[tiab]). For the Scopus search, the search strategy was adapted to database-specific indexing terms.

The studies were selected on the basis of their relevance to pulse antifungal regimens, treatment efficacy, safety outcomes, comparative effectiveness, and practical considerations related to disease management. In addition, reference lists of relevant articles were reviewed to identify further relevant publications. The objective of the search was to provide a comprehensive narrative overview of the available evidence rather than to perform a formal systematic review or meta-analysis. The final search was conducted in April 2026.

## Antifungal pulse regimens

**Fig. 1:**
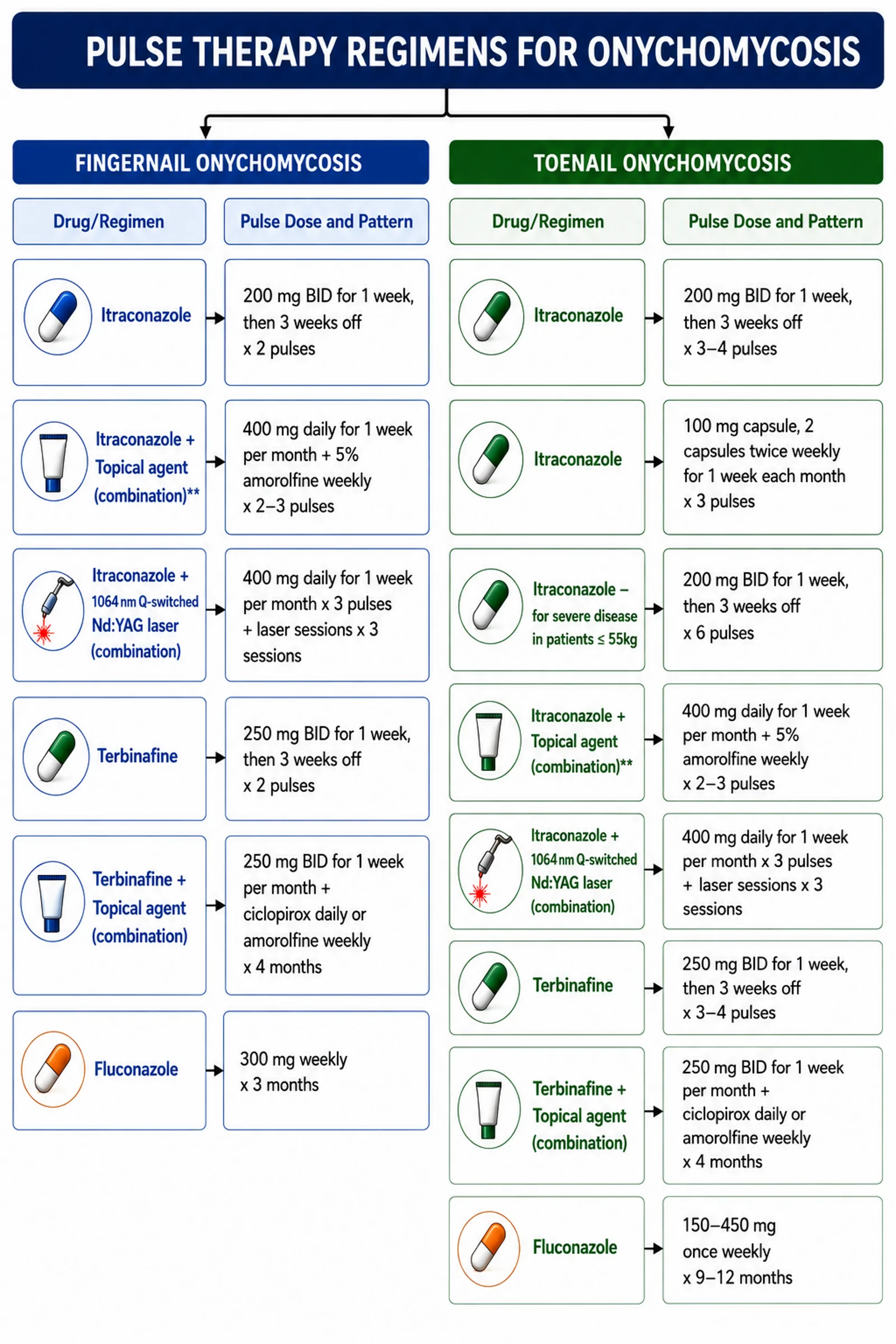
Pulse antifungal regimens used in onychomycosis. **Site of infection not specified

Several antifungal pulse regimens have been investigated for the treatment of onychomycosis, with variations in dose, frequency, and duration depending on the antifungal used and whether fingernails or toenails were affected (Fig. 1). The rationale underlying pulse therapy is the prolonged retention of the antifungals within the nail plate, which permits intermittent administration while maintaining therapeutic drug concentrations in the target tissue.

Itraconazole pulse therapy is the most extensively studied intermittent regimen. The conventional protocol consists of itraconazole 200 mg administered twice daily for 1 week, followed by a 3-week drug-free interval, constituting a single pulse cycle. Typically, two such pulse cycles are administered for fingernail onychomycosis and three to four cycles for toenail infection [9, 17–19, 25, 33–37]. Although this remains the most widely used regimen, several variations have also been explored. These include lower-intensity pulse schedules, extended pulse regimens comprising up to six cycles for severe disease, and combination approaches incorporating topical antifungal agents such as amorolfine [9, 10, 34, 37]. Itraconazole pulse therapy has also been evaluated in comparison with continuous terbinafine therapy and in combination with adjunctive laser-based treatments [17–19, 35].

Pulse terbinafine therapy generally involves administration of a higher daily dose over a short treatment period, most commonly terbinafine 250 mg twice daily for 1 week followed by a 3-week drug-free interval [9, 11, 25, 27, 33, 36, 38, 39]. Similar to itraconazole, two pulse cycles are commonly used for fingernail disease and three to four cycles for toenail involvement, although additional cycles may be administered according to clinical response [11, 12, 36]. Most comparative studies evaluating pulse terbinafine have employed this regimen, including randomized controlled trials comparing pulse and continuous terbinafine therapy, as well as studies examining combinations with topical antifungal agents [9, 11, 12, 38, 39].

Fluconazole is generally administered using a weekly intermittent schedule that resembles pulse therapy. Doses ranging from 150 to 450 mg once weekly for 9–12 months have been reported for dermatophyte toenail onychomycosis [25]. Although less extensively studied than terbinafine or itraconazole, fluconazole has been included in comparative trials evaluating systemic pulse treatment strategies for both fingernail and toenail onychomycosis [40].

Overall, considerable heterogeneity exists among published pulse regimens with respect to dose, treatment duration, and combination strategies. Nevertheless, monthly pulse itraconazole and terbinafine regimens remain the most commonly investigated approaches and constitute the basis of most available efficacy and safety data. Figure 1 summarizes the principal pulse regimens and treatment combinations evaluated in the literature.

## Mechanism of action and rationale for pulse therapy

The efficacy of pulse antifungal therapy is based on both the antifungal mechanisms of the agents used and their favorable pharmacokinetic properties within the nail unit. The two principal systemic agents used in onychomycosis, terbinafine and itraconazole, exert their antifungal activity by disrupting ergosterol biosynthesis, an essential component of the fungal cell membrane.

Terbinafine, an allylamine antifungal, inhibits the enzyme squalene epoxidase, resulting in intracellular accumulation of squalene and depletion of ergosterol. This disruption of fungal cell membrane synthesis ultimately leads to fungal cell death. In contrast, itraconazole, a triazole antifungal, inhibits 14α-demethylase, a cytochrome P450-dependent enzyme involved in ergosterol synthesis, thereby impairing fungal cell membrane formation and function [9, 33, 40]. Because these agents target different steps within the same biosynthetic pathway, sequential treatment strategies combining terbinafine and itraconazole have been investigated with the aim of enhancing antifungal activity through complementary mechanisms of action [33].

Several topical agents have also been used as adjuncts to systemic pulse therapy. Ciclopirox olamine exerts antifungal activity through disruption of multiple cellular metabolic processes, including transmembrane transport mechanisms [11], whereas amorolfine inhibits Δ14-reductase and Δ7–8-isomerase, thereby interfering with ergosterol synthesis through a mechanism distinct from that of both terbinafine and itraconazole [9].

The clinical rationale for pulse therapy is largely pharmacokinetic. Both terbinafine and itraconazole accumulate within the keratinized tissues and persist in the nail plate for prolonged periods after discontinuation of treatment [21]. The therapeutic concentrations may remain detectable for weeks to several months following the administration, allowing the antifungal activity to continue during drug-free intervals [22]. This prolonged tissue retention forms the basis of pulse dosing schedules, in which brief periods of high-dose treatment are followed by treatment-free intervals without a complete loss of antifungal activity. Consequently, pulse regimens may reduce cumulative drug exposure while maintaining efficacy, potentially improving convenience, adherence, and cost-effectiveness in appropriately selected patients [25].

## Treatment cost

One of the important practical advantages of pulse antifungal therapy is the potential reduction in the overall treatment costs through decreased cumulative drug exposure [38, 40]. Because pulse regimens involve intermittent administration rather than continuous daily treatment, the total quantity of drug required is generally lower, which may translate into lower direct medication costs. Several studies have reported that pulse terbinafine regimens are less expensive than continuous terbinafine therapy, while itraconazole pulse therapy is generally associated with higher treatment costs than terbinafine-based regimens [33, 38].

Reports from various countries similarly indicate that pulse therapy may result in cost savings compared with continuous treatment, although the magnitude of these savings varies considerably according to the local drug pricing, healthcare systems, and treatment protocols [11, 36]. In addition to reducing medication costs, pulse regimens may improve convenience and adherence by decreasing the duration of active treatment, potentially reducing the overall burden of therapy for some patients.

Nonetheless, the cost comparisons should be interpreted cautiously because available data are derived from studies conducted in different countries and time periods, and formal pharmacoeconomic analyses remain limited. Moreover, while pulse therapy may represent a cost-effective option in selected settings, its economic advantages are likely to be context-dependent.

## Efficacy data

The efficacy of antifungal pulse therapy in onychomycosis varies according to the antifungal agent used, infecting organism, disease severity, treatment schedule, and outcome assessed (Table 1). Overall, the available evidence supports pulse therapy as an effective treatment strategy for many patients with onychomycosis; however, important differences do exist among the various regimens studied.

**Table 1:** Summary of efficacy studies evaluating pulse antifungal regimens for onychomycosis

Study; site of infection	Regimens compared	Key results	Main conclusion	Citation
Meta-analysis				
Gupta et al. 2020; Dermatophyte toenail (meta-analysis)	Pulse vs. continuous terbinafine/itraconazole (various durations)	Mycological cure similar between pulse/continuous for both drugs; 24-week continuous terbinafine ranked highest overall	Pulse and continuous similar in efficacy/safety	[25]
Pulse vs. continuous terbinafine				
Warshaw et al. 2005; Dermatophyte toenail	Pulse terbinafine 500 mg/day x 1week/month, 3 pulses vs. continuous terbinafine 250 mg daily x 12 weeks	Clinical cure of target toenail at 18 months: 29.3% vs. 44.6% (*p* = 0.007); Complete cure of all target toenail: 28.0% vs. 40.5% (*p* = 0.02)	Continuous therapy superior to pulse therapy	[38]
Yadav et al. 2015; Toenail dermatophytosis	Continuous terbinafine (250 mg/day x 12 weeks) vs. pulse terbinafine (500 mg/day x 1 week/month x 3 months)	Clinical and mycological cure slightly higher with continuous therapy than pulse but not statistically significant	Pulse dosing is as efficacious as continuous therapy	[39]
Saleem et al. 2023; Dermatophyte toenail	Pulse terbinafine 250 mg BID x 1 week on/3 weeks off (3 pulses) vs. continuous terbinafine 250 mg daily x 12 weeks	Week 12: Clinical cure 76.67% with continuous terbinafine vs. 26.67% with pulse terbinafine (*p* < 0.001)	Continuous terbinafine was significantly more effective than high-dose short pulse terbinafine	[27]
Pulse itraconazole vs. continuous therapy				
Gupta et al. 2006; Dermatophyte toenail in diabetic patients	Pulse itraconazole 200 mg BID x 1week/month x 3 pulses vs. continuous terbinafine 250 mg daily x 12 weeks	Mycologic cure: 88.2% vs. 79.3% (NS)	Both pulse itraconazole and continuous terbinafine are comparable in efficacy	[26]
Ali et al. 2022; Onychomycosis	Itraconazole pulse 200 mg BID x 1 week on/3 weeks off vs. continuous terbinafine 250 mg daily x 12 weeks	End of study, clinical + mycologic responder composite: 86.0% for itraconazole pulse vs. 64.0% for continuous terbinafine (*p* < 0.011)	Pulse itraconazole outperformed continuous terbinafine	[35]
Rahmat et al. 2024; Dermatophyte toenail	Pulse itraconazole (2 capsule 100 mg twice weekly x 1week/month x 3 months) vs. continuous itraconazole (100 mg/day x 12 weeks)	Mycologic cure: 83.3% vs. 76.7% (NS)	Pulse and continuous itraconazole therapies have comparable efficacies	[34]
Alternative pulse regimens				
Ranawaka et al. 2016; Non-dermatophyte onychomycosis (fingers/toes)	Itraconazole pulse 400 mg/day x 7 days/month vs. Terbinafine pulse 500 mg/day x 7 days/month (2 pulses for fingers, 3 pulses for toes)	End of therapy: 9.2% vs. 2.0% clinical cure (*p* < 0.05) At 12 months: 65.1% vs. 54.6% clinical cure (NS); Recurrence: 6.5% vs. 4.1% (NS)	Both regimens have partially effective long term clinical cure; early advantage with Itraconazole	[36]
Zhang et al. 2018; Severe toenail onychomycosis	Standard itraconazole pulse therapy (200 mg BID x 1 week/month x 3 months) vs. 6 pulse itraconazole therapy (200–400 mg/day x 1 week/month x 6 months)	Complete cure rate: 32.43% (3 pulses) vs. 75% (6 pulses) (*p* < 0.001)	6 pulses significantly more effective in severe cases (> 50% involvement); 200 mg/day (low dose) is sufficient for patients ≤ 55 kg	[37]
Taha et al. 2022; Mixed nails	Pulse itraconazole 400 mg/day x 1 week/month vs. pulse terbinafine 500 mg/day x 1 week/month vs. weekly Fluconazole 300 mg	At 6-month follow-up, clinical cure, mycologic cure, complete cure with Itraconazole: 80%, 70%, 70%; Terbinafine: 60%, 55%, 55%; Fluconazole: 50%, 35%, 35%; No statistically significant differences	All 3 regimens are effective; numerically highest cure with itraconazole pulses and lowest with fluconazole	[40]
Combination/sequential therapy studies				
Gupta et al. 2001; Dermatophyte toenail	2 itraconazole pulses (200 mg BID x 1 week) followed by 1–2 terbinafine pulses (250 mg BID x 1 week) versus 3–4 terbinafine pulses alone	Mycological cure 72.0% vs. 48.9%; Clinical cure 56.0% vs. 38.9%; Complete cure 52.0% vs. 32.2%; Effective therapy 65.3% vs. 5.6%	Sequential itraconazole-terbinafine therapy outperformed terbinafine pulses alone on all cure endpoints	[33]
Rigopoulos et al. 2003; *Candida* fingernail onychomycosis	2 pulse itraconazole (200 mg BID x 1 week/month x 2 months) + 5% amorolfine lacquer (1 per week x 6 months) vs. 3 pulse itraconazole monotherapy (200 mg BID x 1week/month x 3 months)	Global cure: 93% (combination) vs. 81% (monotherapy) (NS)	Combination therapy is at least as effective (and cost effective) than monotherapy while reducing systemic drug exposure	[10]
Jaiswal et al. 2007; Dermatophyte onychomycosis	Pulse terbinafine monotherapy (250 mg BID x 1week/month x 4 months) vs. pulse terbinafine + topical ciclopirox olamine 8% applied once daily vs. pulse terbinafine + topical amorolfine HCl 5% applied once weekly	Clinical cure: 71.73% vs. 82.60% vs. 73.91% (NS); Mycologic cure: 88.9% vs. 88.9% vs. 85.7% (NS)	Pulse terbinafine is effective; combinations with topicals are not significantly better	[11]
Phutane et al. 2025; Onychomycosis	Pulse terbinafine 250 mg BID x 1 week/month x 3 months + topical amorolfine 5% once a week x 3 months vs. pulse itraconazole 200 mg BID x 1 week/month x 3 months + topical amorolfine 5% once a week x 3 months	Mycologic cure: 78.57% vs. 88.88% (NS); Complete cure: 26.31% vs. 21.05% (NS)	Combination therapies are effective and comparable, with similar cure rates	[9]
Laser-based adjunctive therapies				
Dash et al. 2025; Onychomycosis	Pulse itraconazole (400 mg/day x 1 week/month x 3 months) vs. 1064 nm Q-switched Nd: YAG laser (3 sessions-on day 0, 30 and 60) vs. Combination of both	Complete cure rate: 66.7%, 74.1%, 80% (NS); All 3 groups showed OSI reduction from baseline (*p* < 0.001)	Combination therapy shows numerically high cure rate but not statistically significant; laser therapy is as effective as pulse itraconazole therapy, can be a safer alternative due to lack of systemic side effects	[19]

Among the pulse regimens, itraconazole has demonstrated the most consistently favorable efficacy profile. Multiple studies have reported clinical and mycological cure rates comparable to those achieved with continuous oral therapy, including in patients with diabetes mellitus and those with mixed nail infections [26, 34, 35]. Furthermore, modified treatment schedules, including lower-intensity dosing regimens and prolonged pulse protocols extending beyond the conventional three-cycle regimen, have shown promising results in selected patient populations, particularly among those with more extensive disease [34, 37]. Collectively, these findings suggest that pulse itraconazole represents a clinically useful alternative when continuous terbinafine is not suitable or contraindicated.

Combination and sequential treatment strategies have also yielded encouraging results. The regimens combining itraconazole and terbinafine pulses, or pulse therapy with topical agents such as amorolfine, have demonstrated cure rates comparable to or numerically higher than those observed with antifungal monotherapy in several studies [9, 10, 33]. Similarly, studies evaluating antifungal pulse therapy in combination with laser-based interventions have reported favorable outcomes, although statistically significant superiority over conventional treatment has not been consistently demonstrated [17–19]. While these findings suggest potential benefits from the multimodal treatment approaches, the available evidence remains limited due to the small sample sizes and heterogeneity in the study designs.

In contrast, evidence supporting pulse terbinafine is less consistent. Although several open-label and combination studies have reported satisfactory clinical outcomes [9, 11], randomized controlled trials directly comparing pulse and continuous terbinafine generally favor continuous therapy, particularly in dermatophyte toenail onychomycosis [27, 38]. Continuous terbinafine has repeatedly demonstrated higher clinical and complete cure rates in these studies, although not all investigations have identified statistically significant differences between treatment strategies [39]. Consequently, while pulse terbinafine may remain a reasonable option in selected patients, current evidence does not support its routine use in place of continuous terbinafine for dermatophyte toenail disease.

Fluconazole has also been evaluated using weekly intermittent dosing schedules that resemble pulse therapy. Although effective in some patients, reported cure rates are generally lower than those observed with terbinafine- or itraconazole-based regimens, supporting its role as a second-line treatment option when first-line therapies are unsuitable or unavailable [40].

Taken together, the available evidence continues to support continuous terbinafine as the most reliable treatment for dermatophyte toenail onychomycosis. Nonetheless, pulse therapy remains a valuable therapeutic strategy in appropriately selected patients. Among currently available pulse regimens, itraconazole appears to have the strongest supporting evidence, whereas modified pulse schedules, prolonged treatment protocols, and combination approaches may further expand the therapeutic utility of pulse antifungal therapy. However, interpretation of the literature is complicated by substantial heterogeneity in study design, treatment regimens, outcome definitions, and duration of follow-up, highlighting the need for further well-designed comparative studies.

## Key safety data

Available evidence suggests that pulse antifungal regimens are generally well tolerated and have safety profiles comparable to those of continuous therapy (Table 2). Across multiple studies evaluating terbinafine, itraconazole, and fluconazole-based regimens, most reported adverse events were mild to moderate in severity and rarely resulted in treatment discontinuation [9, 11, 25, 33, 36, 38].

**Table 2:** Summary of safety outcomes reported in studies evaluating pulse antifungal regimens for onychomycosis

Study; site of infection	Regimens compared	Adverse event (AE) data	Main conclusion	Citation
Meta-analysis				
Gupta et al. 2020; Dermatophyte toenail	Pulse vs. continuous terbinafine/itraconazole (various durations)	No significant difference in likelihood of AEs between the groups	Pulse and continuous regimens offer comparable safety profiles	[25]
Pulse vs. continuous terbinafine				
Warshaw et al. 2005; Dermatophyte toenail	Pulse terbinafine 500 mg/day x 1week/month, 3 pulses vs. continuous terbinafine 250 mg daily x 12 weeks	Common side effects like diarrhoea, nausea, flatulence; 18 patience discontinued due to other AEs	No significant difference in tolerability between the two groups	[38]
Yadav et al. 2015; Toenail dermatophytosis	Continuous terbinafine 250 mg/day x 12 weeks vs. pulse terbinafine 500 mg/day x 1 week/month x 3 months	10.5% of total patients (5 from continuous, 3 from pulse group) had mild gastric discomfort; no severe biochemical abnormalities	Both regimens were well tolerated, no significant difference in safety or laboratory abnormalities	[39]
Saleem et al. 2023; Dermatophyte toenail	Pulse terbinafine 250 mg BID x 1 week on/3 weeks off (3 pulses) vs. continuous terbinafine 250 mg daily x 12 weeks	Specific AE data not reported	Nil	[27]
Pulse itraconazole vs. continuous therapy				
Gupta et al. 2006; Dermatophyte toenail in diabetic patients	Pulse itraconazole 200 mg BID x 1week/month x 3 pulses vs. continuous terbinafine 250 mg daily x 12 weeks	Gastrointestinal problems reported by 3 patients in Itraconazole group	No serious AEs, no interactions recorded with concomitant antidiabetes medications	[26]
Ali et al. 2022; Onychomycosis	Itraconazole pulse 200 mg BID x 1 week on/3 weeks off vs. continuous terbinafine 250 mg daily x 12 weeks	No significant adverse events; headache and nausea were shared minor symptoms	Both regimens were safe and well tolerated and no drug interactions	[35]
Rahmat et al. 2024; Dermatophyte toenail	Pulse itraconazole 2 capsules 100 mg twice weekly x 1 week/month x 3 months vs. continuous itraconazole 100 mg/day x 12 weeks	Liver and renal function tests were monitored throughout the study to evaluate safety of modified dosing	No significant findings were reported	[34]
Alternative pulse regimens				
Ranawaka et al. 2015; Non-dermatophyte onychomycosis (fingers/toes)	Itraconazole pulse 400 mg/day x 7days/month vs. terbinafine pulse 500 mg/day x 7 days/month (2 pulses fingers, 3 pulses toes)	2 patients in itraconazole group discontinued due to worsened gastritis; nausea was also reported	Regimens were generally safe; GI distress was the primary cause for treatment cessation	[36]
Zhang et al. 2018; Severe toenail onychomycosis	Standard itraconazole pulse therapy 200 mg BID x 1 week/month x 3 months vs. 6 pulse itraconazole therapy 200–400 mg/day x 1 week/month x 6 months	Gastrointestinal discomfort and headache reported; 9 patients had transient increase in conjugated bilirubin	Most AEs were mild and transient; no significant safety differences between 3 pulses and 6 pulses	[37]
Taha et al. 2022; Onychomycosis - mixed infection	Pulse itraconazole 400 mg/day x 1 week/month vs. pulse terbinafine 500 mg/day x 1 week/month vs. weekly Fluconazole 300 mg	All 60 patients completed the treatment protocol	Suggests high tolerability across all regimens	[40]
Combination/sequential therapy studies				
Gupta et al. 2001; Dermatophyte toenail	2 itraconazole pulses (200 mg BID x 1 week) followed by 1–2 terbinafine pulses (250 mg BID x 1 week) vs. 3–4 terbinafine pulses alone	Reported AEs in 14.8% of sequential itraconazole-terbinafine group vs. 23.2% in pulse terbinafine alone group	Both regimens were well tolerated, with low permanent discontinuation rates (2.5% vs. 2.1%) and no new safety concerns	[33]
Rigopoulos et al. 2003; *Candida* fingernail onychomycosis	2 pulse itraconazole (200 mg BID x 1 week/month x 2 months) + 5% amorolfine lacquer 1 per week x 6 months vs. 3 pulse itraconazole monotherapy 200 mg BID x 1week/month x 3 months	Most frequent complaints were nausea and abdominal pain	Gastrointestinal disturbance, mild to moderate, is usually the most common side effect of triazole-based treatment	[10]
Jaiswal et al. 2007; Dermatophyte onychomycosis	Pulse terbinafine monotherapy (250 mg BID x 1 week/month x 4 months) vs. pulse terbinafine + topical ciclopirox olamine 8% applied once daily vs. pulse terbinafine + topical amorolfine HCl 5% applied once weekly	AEs observed in 13% of patients (diarrhoea, headache, dyspnea, taste disturbance, pruritis, abnormal liver enzymes); only 1 participant discontinued the study because of liver enzyme abnormality (returned to normal in 4 weeks)	All AEs resolved with time	[11]
Phutane et al. 2025; Onychomycosis	Pulse terbinafine 250 mg BID x 1 week/month x 3 months + topical amorolfine 5% once a week x 3 months vs. pulse itraconazole 200 mg BID x 1 week/month x 3 months + topical amorolfine 5% once a week x 3 months	Reported AEs were nausea, vomiting, gastrointestinal distress, diarrhoea, abdominal pain and headache	No significant differences in AEs between the two groups; all AEs of mild to moderate severity and reversible	[9]
Laser-based adjunctive therapies				
Dash et al. 2025; Onychomycosis	Pulse itraconazole (400 mg/day x 1 week/month x 3 months vs. 1064 nm Q-switched Nd: YAG laser (3 sessions-on day 0, 30 and 60) vs. combination of both	Reported gastrointestinal and taste disturbances in oral itraconazole patients; patients in laser/combination groups complained of pain during procedure	Oral therapy causes mild gastrointestinal issues, and laser procedure is associated with transient pain	[19]

Gastrointestinal symptoms, including nausea, abdominal discomfort, diarrhoea, and dyspepsia, were the most frequently reported adverse effects, followed by headache and occasional taste disturbances [9, 11, 36]. These events were typically transient and self-limiting. Rates of treatment discontinuation due to adverse effects were generally low for both pulse and continuous regimens, indicating good overall tolerability [11, 33, 36, 38].

Comparative studies evaluating pulse itraconazole and pulse terbinafine have not demonstrated clinically meaningful differences in the frequency or severity of adverse events [36]. Similarly, modified pulse schedules and prolonged pulse regimens have not been associated with a significant increase in treatment-related toxicity, suggesting that extending or adjusting pulse strategies does not substantially compromise tolerability [34, 37]. Routine clinical practice generally includes assessment of hepatic function before initiating systemic antifungal therapy, with periodic monitoring in selected patients receiving prolonged treatment or those with relevant comorbidities [21, 34, 41].

Evidence from studies involving specific patient populations, including individuals with diabetes mellitus, has not identified any major safety concerns or clinically significant drug interactions attributable to pulse therapy [26]. In addition, a meta-analysis comparing pulse and continuous antifungal regimens found no significant differences in the overall risk of adverse events, providing additional support for the comparable safety profiles of the two treatment approaches [25].

Combination strategies incorporating topical antifungal agents or adjunctive modalities, such as laser therapy, have similarly demonstrated favorable safety profiles. The reported adverse effects were generally mild and consistent with those expected from the individual treatment components, with no clear evidence of increased toxicity resulting from the combination therapy [9–11, 19, 34].

Overall, the current evidence indicates that pulse antifungal therapy is a safe and well-tolerated treatment option for onychomycosis. Although pulse regimens reduce cumulative drug exposure, available data suggest that their principal advantage lies in maintaining comparable tolerability while potentially improving the convenience and reducing treatment burden. Further long-term studies are needed to determine whether the reduced cumulative exposure translates into clinically meaningful reductions in adverse drug reactions.

## Conclusion

Antifungal pulse therapy represents an important treatment strategy for onychomycosis and has demonstrated efficacy across a variety of clinical settings. However, the effectiveness of pulse regimens varies according to the antifungal agent used, infecting organism, disease severity, and treatment protocol employed. Among the currently available regimens, continuous oral terbinafine remains the most consistently effective treatment for dermatophyte toenail onychomycosis and is supported by data from randomized controlled trials, long-term follow-up studies, and contemporary clinical guidelines.

Among the pulse regimens, itraconazole has the strongest supporting evidence and appears to provide outcomes comparable to continuous therapy in selected patient populations, including those with mixed infections and certain comorbidities. In contrast, evidence supporting pulse terbinafine is less consistent, with several comparative studies favoring continuous administration, particularly in dermatophyte toenail disease. Modified pulse schedules, prolonged pulses, and combination approaches incorporating topical antifungals may further expand the therapeutic utility of pulse therapy, although additional evidence is required to define their optimal role in clinical practice.

From a safety perspective, pulse and continuous antifungal regimens appear to have broadly comparable tolerability profiles. Most reported adverse events are mild, self-limiting, and predominantly gastrointestinal in nature, while serious treatment-related toxicity remains uncommon. Although pulse regimens reduce the cumulative drug exposure, the extent to which this translates into clinically meaningful reductions in long-term adverse effects remains uncertain.

Taken together, the current evidence supports continuous terbinafine as the preferred first-line systemic treatment for dermatophyte onychomycosis, while pulse itraconazole remains a useful alternative in appropriately selected patients. Treatment decisions should be individualized according to the causative organism, extent of nail involvement, patient comorbidities, treatment preferences, and practical considerations such as cost and adherence. Despite a substantial body of literature, direct comparisons between pulse and continuous regimens remain limited by heterogeneity in study design, treatment schedules, outcome definitions, and duration of follow-up. Therefore, well-designed randomized trials employing standardized outcome measures and long-term assessment of recurrence are needed to better define the role of pulse therapy and inform evidence-based treatment algorithms.

## Data Availability

No datasets were generated or analysed during the current study.
